# Development of a novel cell line‐derived xenograft model of primary herpesvirus 8‐unrelated effusion large B‐cell lymphoma and antitumor activity of birabresib *in vitro* and *in vivo*

**DOI:** 10.1002/cam4.4394

**Published:** 2021-11-24

**Authors:** Tomohiro Nishimori, Tomonori Higuchi, Yumiko Hashida, Takako Ujihara, Ayuko Taniguchi, Fumiya Ogasawara, Naoya Kitamura, Ichiro Murakami, Kensuke Kojima, Masanori Daibata

**Affiliations:** ^1^ Department of Microbiology and Infection Kochi Medical School Kochi University Nankoku Japan; ^2^ Science Research Center Kochi University Nankoku Japan; ^3^ Department of Hematology Kochi Medical School Kochi University Nankoku Japan; ^4^ Department of Oral and Maxillofacial Surgery Kochi Medical School Kochi University Nankoku Japan; ^5^ Department of Pathology Kochi Medical School Kochi University Nankoku Japan

**Keywords:** clinical observations, experimental therapeutics, lymphoma, mouse model

## Abstract

**Background:**

Primary human herpesvirus 8 (HHV8)‐unrelated effusion large B‐cell lymphoma is a clinical disease entity distinct from HHV8‐positive primary effusion lymphoma (PEL). However, the lack of experimental HHV8‐unrelated effusion large B‐cell lymphoma models continues to hinder the pathophysiologic and therapeutic investigations of this disorder.

**Methods:**

The lymphoma cells were obtained from the pleural effusion of a patient with primary HHV8‐unrelated effusion large B‐cell lymphoma and cultured *in vitro*.

**Results:**

We established a novel HHV8‐unrelated effusion large B‐cell lymphoma cell line, designated Pell‐1, carrying a *c‐MYC* rearrangement with features distinct from those of HHV8‐positive PEL. Moreover, we developed an HHV8‐unrelated effusion large B‐cell lymphoma cell line‐derived xenograft model. Pell‐1 cells induced profuse lymphomatous ascites and subsequently formed intra‐abdominal tumors after intraperitoneal implantation into irradiated nonobese diabetic/severe combined immunodeficient mice. Thus, this xenograft mouse model mimicked the clinical phenomena observed in patients and recapitulated the sequential stages of aggressive HHV8‐unrelated effusion large B‐cell lymphoma. The bromodomain and extraterminal domain (BET) inhibitors JQ1 and birabresib (MK‐8628/OTX015) reduced the proliferation of Pell‐1 cells *in vitro* through the induction of cell cycle arrest and apoptosis. The antitumor effect of BET inhibition was also demonstrated *in vivo*, as birabresib significantly reduced ascites and suppressed tumor progression without apparent adverse effects in the xenografted mice.

**Conclusion:**

These preclinical findings suggest the therapeutic potential of targeting c‐MYC through BET inhibition in HHV8‐unrelated effusion large B‐cell lymphoma.

## Graphical abstract

A new herpesvirus 8 (HHV8)‐unrelated effusion large B‐cell lymphoma cell line, Pell‐1, with a *c‐MYC* rearrangement induced profuse lymphomatous ascites and subsequently formed intra‐abdominal tumors after intraperitoneal implantation into irradiated NOD/SCID mice. This xenograft model mimicked the clinical phenomena observed in patients and recapitulated the sequential stages of aggressive HHV8‐unrelated effusion large B‐cell lymphoma. The BET inhibitor birabresib significantly suppressed effusion formation and tumor progression in mice.

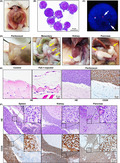

## 1 INTRODUCTION

Patients with primary effusion lymphoma (PEL) typically present with lymphomatous effusion in a body cavity, but occasionally with an extracavitary mass or both.^1^, ^2^, ^3^ PELs are universally associated with human herpesvirus 8 (HHV8) and are often coinfected with Epstein–Barr virus (EBV), especially in the setting of human immunodeficiency virus (HIV) infection.^1^ HHV8, rather than EBV and HIV, is recognized to play an indispensable role in the development of PEL.^4^, ^5^ Cases of HHV8‐negative primary lymphomatous effusions have been reported and these are referred to collectively as primary HHV8‐unrelated effusion large B‐cell lymphoma, primary HHV8‐negative effusion‐based lymphoma, or HHV8‐unrelated PEL‐like lymphoma. Intriguingly, patients of East Asian origin—especially Japanese—account for approximately 60% of cases of reported HHV8‐unrelated effusion large B‐cell lymphomas.^6^, ^7^, ^8^, ^9^, ^10^, ^11^, ^12^, ^13^ HHV8‐unrelated effusion large B‐cell lymphoma is often seen in individuals with underlying adverse medical conditions, such as liver cirrhosis and congestive heart failure, which lead to fluid overload in body cavities.^7^, ^8^ HHV8‐unrelated effusion large B‐cell lymphoma exhibits an overlapping clinical presentation with PEL, but with a distinct immunophenotype (usually positive for B‐cell‐associated antigens) and lack of association with HIV.^6^, ^7^, ^8^, ^9^, ^10^ Clinical outcomes of cases of HHV8‐unrelated effusion large B‐cell lymphoma are relatively favorable compared with cases of PEL, although some have an aggressive clinical course with poor prognosis.^6^, ^11^, ^12^, ^13^ Cases of PEL consistently lack the recurrent cytogenetic abnormalities commonly seen in B‐cell malignancies, such as *c‐MYC* rearrangement.^3^, ^14^ By contrast, *c‐MYC* alterations are associated with a significant fraction of HHV8‐unrelated effusion large B‐cell lymphoma.^6^, ^12^, ^15^ Thus, although HHV8‐unrelated effusion large B‐cell lymphoma has not been included as a clinical entity in the 2017 World Health Organization classification, it appears to differ biologically from HHV8‐positive PEL and remains an incompletely characterized disease entity. However, the rarity of HHV8‐unrelated effusion large B‐cell lymphoma, coupled with a lack of suitable study systems, might have hindered investigations of its pathogenesis and therapeutic strategies for this disease.

Cell lines provide invaluable tools for research on rare diseases, by which unlimited supplies of tumor cells can be studied repeatedly and extensively. Although numerous HHV8‐positive PEL cell lines have been reported,^16^, ^17^ only a few lines derived from HHV8‐unrelated primary lymphomatous effusions have been reported.^18^, ^19^ However, most of these cell lines have not been authenticated genetically against the primary tumor. Hence, there is a continued need for validated patient‐derived HHV8‐unrelated effusion large B‐cell lymphoma cell lines to increase our understanding of this unique form of lymphoma and to provide tools for the development of effective strategies. While readily amenable to experimentation, *in vitro* culture does not fully recapitulate all features of the tumor, thus motivatiing the development of an *in vivo* HHV8‐unrelated effusion large B‐cell lymphoma model that presents lymphomatous effusion in the body cavity.

Herein, we describe a fully characterized HHV8‐unrelated effusion large B‐cell lymphoma cell line carrying a *c‐MYC* rearrangement, designated Pell‐1. We also established a novel HHV8‐unrelated effusion large B‐cell lymphoma cell line‐derived xenograft (CDX) model employing nonobese diabetic/severe combined immunodeficient (NOD/SCID) mice, in which lymphoma cells proliferate steadily while producing profuse lymphomatous effusions in the peritoneal cavity. Using this Pell‐1 model, we showed the potential efficacy of c‐MYC‐targeted therapy for HHV8‐unrelated effusion large B‐cell lymphoma using bromodomain and extraterminal domain (BET) inhibitors *in vitro* and *in vivo*.

## 2 MATERIALS AND METHODS

### 2.1 Case history and cell culture

A 76‐year‐old Japanese man, who had been suffering from long‐standing alcohol‐induced liver cirrhosis over the past 25 years, presented with bilateral pleural effusions. No evidence of mass lesions or lymphadenopathy was found. Cytology of the effusion revealed medium‐to‐large‐sized atypical lymphoid cells without prominent vacuoles (Figure S1A). The specimen showed atypical tumor cells with the phenotype of CD20^+^, CD10^–^, BCL6^+^, MUM1^+^, indicating a nongerminal center B‐cell type lymphoma. The cells were negative for CD138. Conventional cytogenetic analysis showed a complex karyotype (Figure S2). Fluorescence in situ hybridization (FISH) analysis showed *c‐MYC* and *BCL6* rearrangements (Figure S1B,C), whereas *BCL2* rearrangement was not detected. The cells were found to be negative for the HHV8 and EBV genomes by polymerase chain reaction (PCR). Immunocytochemical staining for HHV8 on cell block specimens also showed a negative result. Serologic testing was negative for HIV. Based on these findings, the patient was diagnosed as having primary HHV8‐unrelated effusion large B‐cell lymphoma. He was treated with rituximab. However, the disease relapsed 6 months later with lymphomatous pleural effusions, and the patient died 10 months after the initial presentation.

Lymphoma cells were collected from pleural effusions at the initial diagnosis. The cells were cultured in RPMI‐1640 medium supplemented with 20% heat‐inactivated fetal calf serum (FCS) without any external stimulation. The cultures were incubated at 37°C in a humidified atmosphere of 5% CO_2_ in the air and fed every 3 days by partial medium replacement. This study was approved by the Ethics Committee of Kochi Medical School, Kochi University, and written informed consent was obtained from the patient at his initial diagnosis.

### 2.2 Characterization of the cell line

The presence of the HHV8 and EBV genomes was examined using PCR with specific primers for the ORF26 (KS330_233_) and *Bam*HI‐W (TC60/TC61) fragments, respectively.^20^, ^21^ Expression of cell surface antigens was studied with fluorescein isothiocyanate‐conjugated monoclonal antibodies using flow cytometry. To evaluate the expression of c‐MYC protein in Pell‐1 cells, immunofluorescent double staining for c‐MYC and CD20 was performed. Cells were fixed with 4% paraformaldehyde, treated with blocking reagents, and reacted with rabbit monoclonal anti‐human c‐MYC (clone D84C12; Cell Signaling Technology) and mouse monoclonal anti‐human CD20 (clone L26; Nichirei Biosciences). Chromosomal analysis and spectral karyotyping (SKY) analysis were performed as described.^22^, ^23^ *c‐MYC* rearrangements were detected in interphase nuclei by FISH analysis using dual‐color, break‐apart rearrangement probes flanking the breakpoint regions of the genes.^24^ Short tandem repeat (STR) DNA fingerprinting was carried out using a GenePrint 10 System (Promega), which allows coamplification and detection of 10 human loci, namely, *TH01*, *D21S11*, *D5S818*, *D13S317*, *D7S820*, *D16S539*, *CSF1PO*, *Amelogenin*, *vWA*, and *TPOX*.

### 2.3 Cell proliferation, apoptosis, and cell cycle analyses

For cell proliferation assays, they were seeded into 96‐well plates (2 × 10^4^ cells/well), and viable cells were counted on an FACSCalibur flow cytometer (Becton Dickinson) by gating out cells stained with propidium iodide. Apoptosis and cell cycle analyses were performed as described.^25^ PEL cell lines used in this study were obtained from American Type Culture Collection (Manassas, VA, USA). The BET inhibitors JQ1 and birabresib (MK‐8628/OTX015) were purchased from Merck KGaA (Darmstadt, Germany) and MedChemExpress (Monmouth Junction, NJ, USA), respectively, and dissolved in dimethyl sulfoxide (DMSO).

### 2.4 Xenograft model

NOD/SCID mice were purchased from Charles River Laboratories (Yokohama, Japan). Pell‐1 cells from culture were washed twice in a serum‐free RPMI‐1640 medium, and aliquots of 3 × 10^7^ cells were resuspended in 1.0 ml of the same medium. Unirradiated or irradiated 6‐week‐old male mice were each injected intraperitoneally with a single aliquot of cells. Irradiated mice received whole‐body irradiation (250 rad) 1 day before injection with the cells. Euthanized mice were subjected to laparotomy and thoracotomy and evaluated for the development of effusions and tumors. Peritoneal lavages were collected using 1.0 ml of phosphate‐buffered saline. Excised organ samples were fixed in 10% formalin and used for histology. All experimental protocols were approved by our Institutional Animal Care and Use Committee in compliance with our institutional guidelines and the National Institutes of Health Guide for the Care and Use of Laboratory Animals (NIH Publications).

### 2.5 Statistical analysis

Mann–Whitney nonparametric *U* tests were used to analyze differences between the pairs of groups. Kruskal–Wallis ANOVA followed by Dunn's multiple comparison tests were used when more than two groups were compared. A *p* value <0.05 was considered significant.

## 3 RESULTS

### 3.1 Generation and characteristics of the Pell‐1 cell line

**FIGURE 1:**
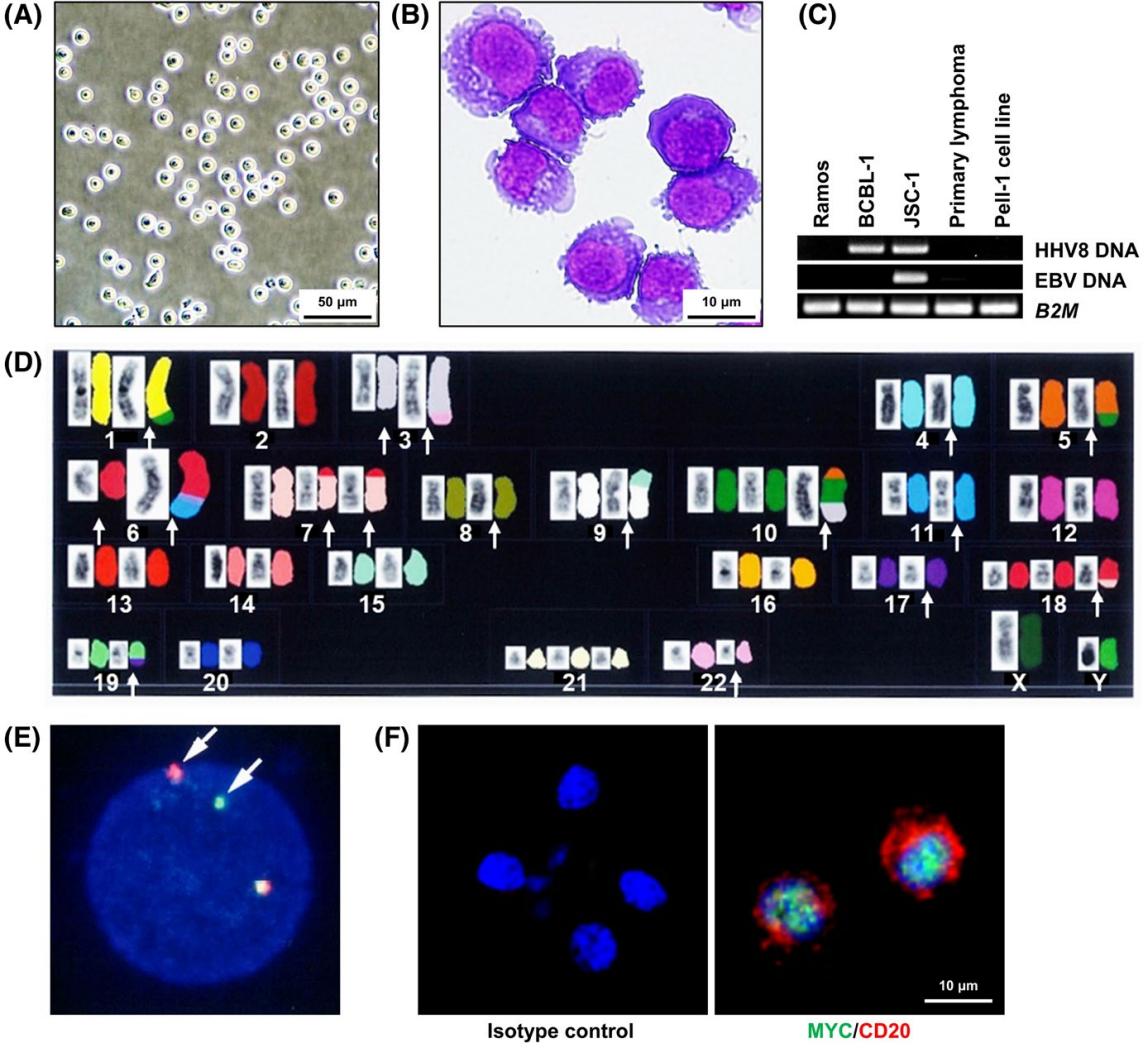
Characteristics of Pell‐1 cells. (A) Phase‐contrast microphotograph of growing Pell‐1 cells. (B) Cytospin preparation of Pell‐1 cells (May–Giemsa staining). (C) PCR analysis for the HHV8 and EBV genomes, showing the absence of the viral DNA in Pell‐1 and primary lymphoma cells. The following cell lines were used as controls: HHV8‐negative and EBV‐negative Burkitt's lymphoma cell line Ramos, HHV8‐positive and EBV‐negative PEL cell line BCBL‐1, and HHV8‐positive and EBV‐positive PEL cell line JSC‐1. The β2‐microglobulin gene (*B2M*) was used as a loading control. (D) Karyotyping by SKY analysis (left side, reverse 4′,6‐diamidino‐2‐phenylindole staining; right side, multicolor fluorescence in situ hybridization). The karyotype from combined G‐banding and SKY analyses was as follows: 50, XY, der(1)inv(1)(p11q21)t(1;10)(q42;?), der(3)del(3)(p?)del(3)(q?), der(3)dup(3)(q21q27)t(3;22)(q27;q11.2), del(4)(q?), der(5)t(5;10)(q22;?), der(6)t(6;11)(q25;q13)inv(6;?)(q25;?), der(7)t(7;18)(p15;q21)del(7)(q?), der(8)(8?::p21→qter), der(9)t(9;15)(p24;q15)inv(9)(p12q13), der(10) t(5;10)(?;q13)t(3;10)(?;q24), der(11)(pter→q23.3::11?), del(17)(p?), der(18)t(7;18), der(19)t(17;19)(?;q13.1). Arrows indicate structural chromosomal abnormalities. (E) FISH analysis for *c*‐*MYC* rearrangement using dual‐color, break‐apart probes, showing split signals for *c*‐*MYC* (arrows). A yellow signal corresponds to the intact locus, whereas separate red (5′ *c*‐*MYC* probe) and green (3′ *c*‐*MYC* probe) signals indicate gene rearrangement. (F) Immunofluorescent double staining for c‐MYC (green) and CD20 (red) proteins in Pell‐1 cells. The nuclei were counterstained with 4′,6‐diamidino‐2‐phenylindole (blue). c‐MYC expression was observed in the nuclei of the CD20‐positive Pell‐1 cells. Alexa Fluor 488‐labeled goat anti‐rabbit IgG and Alexa Fluor 594‐labeled goat anti‐mouse IgG was used as the secondary antibodies. Results of double staining using isotype controls for anti‐c‐MYC and anti‐CD20 antibodies are also shown

The cells began to proliferate 4 weeks after the initiation of culture and then could be regularly passaged in RPMI‐1640 medium supplemented with 20% FCS. The cells were frozen in CELLBANKER™ (Takara Bio) and could be revived after storage in liquid nitrogen. Pell‐1 cells (Figure 1A) grew in single‐cell suspensions with a doubling time of 28 h. The cell line was composed of medium‐to‐large‐sized cells (Figure 1B). The nuclei were round or slightly irregular with slightly coarse chromatin and each had a single nucleolus. No prominent vacuoles were observed in the cytoplasm or nucleus. The morphology of Pell‐1 cells closely resembled that of primary lymphoma cells. The absence of HHV8 and EBV was confirmed using PCR (Figure 1C).

The immunophenotypes of Pell‐1 cells were virtually identical to the primary tumor cells. They were positive for B‐cell markers including CD19, CD20, CD22, and CD79a but negative for CD10, CD23, CD138, and T‐cell markers. The karyotype of Pell‐1 cells after 20 passages showed a close resemblance to that of the primary lymphoma cells (Figure S1), indicating that they were indeed derived from these cells. Karyotypes combining G‐banding and SKY analyses are shown in Figure 1D. FISH analysis revealed *c‐MYC* rearrangement in all Pell‐1 cells analyzed (Figure 1E). *BCL6* rearrangement was also detected. Immunostaining analysis demonstrated that c‐MYC was consistently expressed in the nuclei of the Pell‐1 cells (Figure 1F).

STR DNA fingerprinting analysis based on genotyping of 10 loci showed that the primary lymphoma cells and Pell‐1 cells shared 100% identity (Figure S3). This also demonstrated that the Pell‐1 cells were indeed derived from the patient's tumor cells.

### 3.2 Cell growth assay of Pell‐1 cell line

**FIGURE 2:**
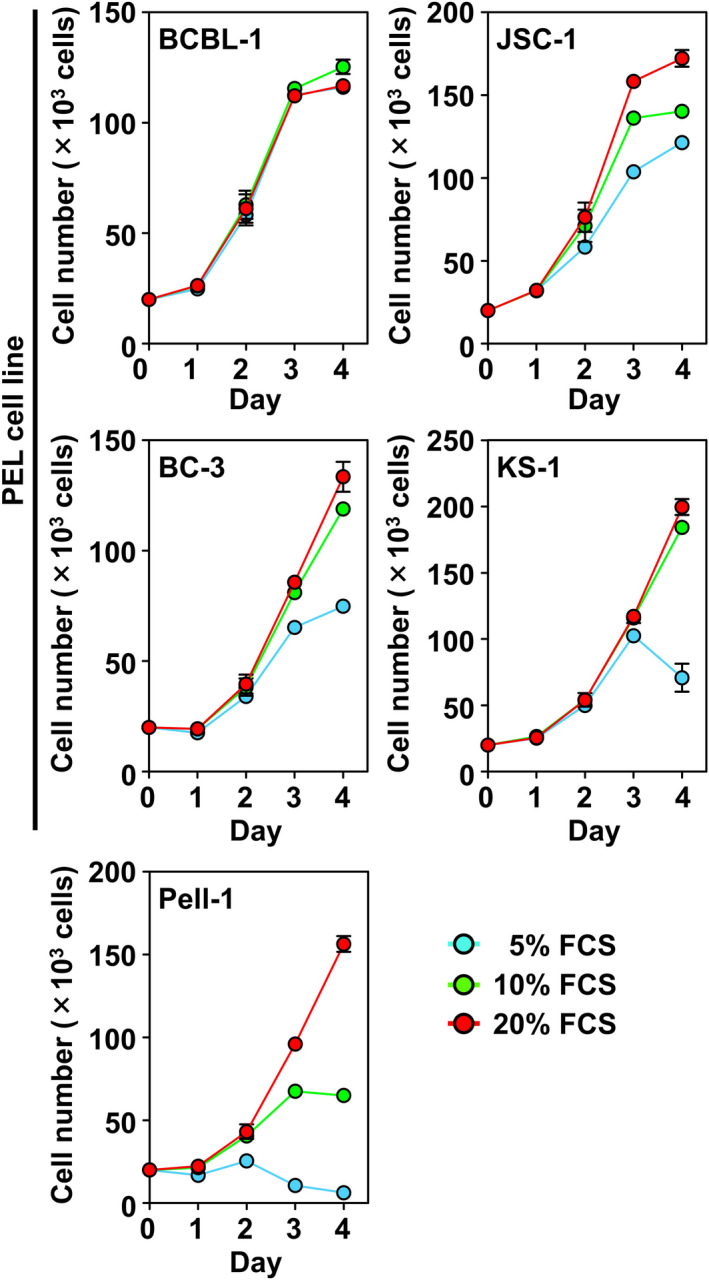
Growth curves of Pell‐1 and HHV8‐positive PEL cell lines. Pell‐1 and PEL cell lines (BCBL‐1, JSC‐1, BC‐3, and KS‐1) were resuspended at a density of 2 × 10^5^ cells/mL in medium supplemented with 20%, 10%, or 5% FCS, and viable cell numbers were counted daily for 4 days. Results from three separate experiments are shown as the mean ± standard error of the mean (SEM; bars)

The growth ability of Pell‐1 cells was compared with that of four HHV8‐positive PEL cell lines (BCBL‐1, JSC‐1, BC‐3, and KS‐1) in RPMI‐1640 medium containing various concentrations of FCS (Figure 2). Pell‐1 cells showed continuous vigorous growth in medium supplemented with 20% FCS. However, this cell line showed decay in cell growth after 3 days of culture in medium with 10% FCS and lost viability in medium with 5% FCS. By contrast, cell growth was observed in all PEL cell lines with 10% FCS and in some PEL cell lines even with 5% FCS.

### 3.3 Establishment of a xenograft model

**FIGURE 3:**
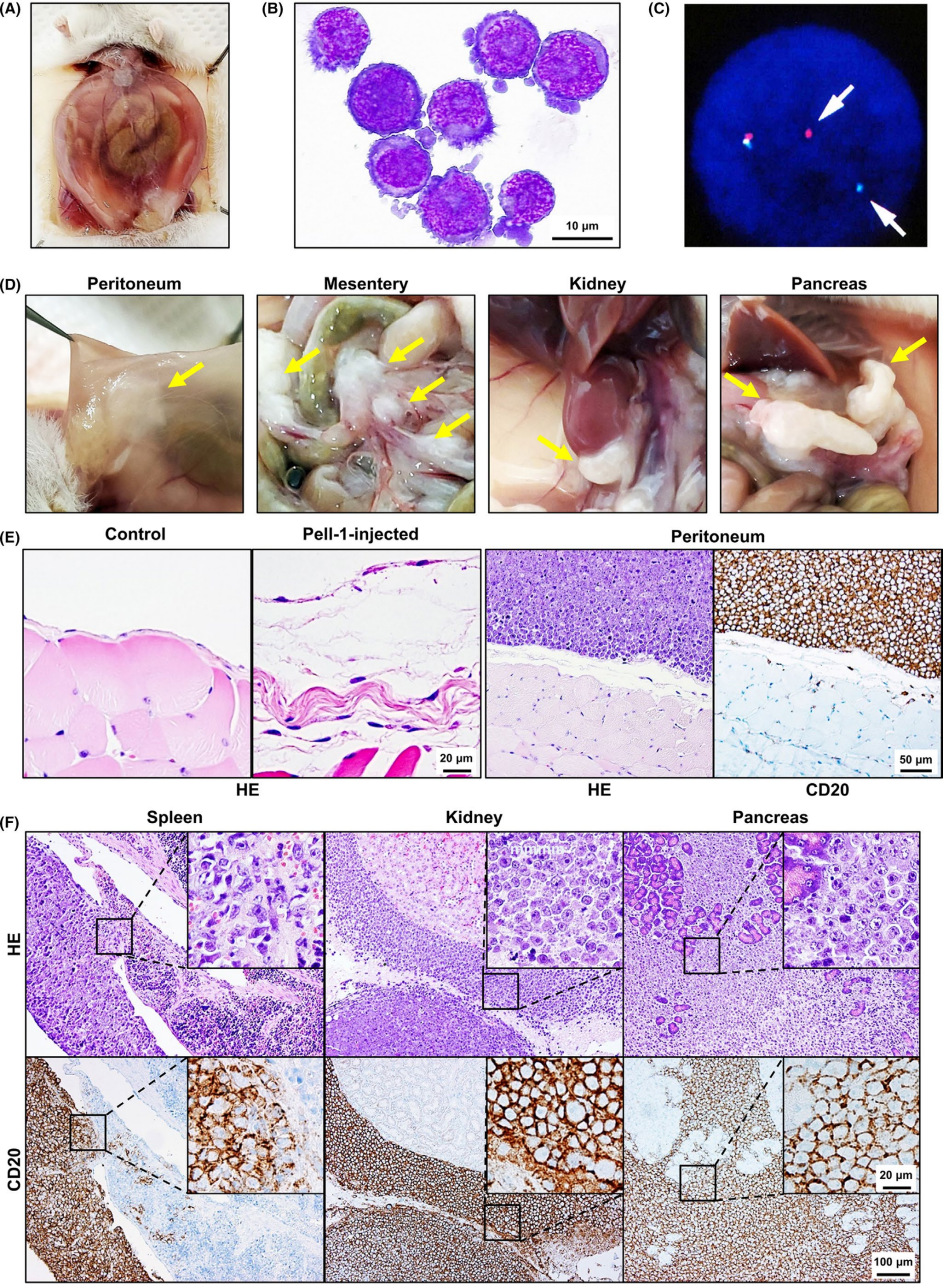
Intraperitoneal xenotransplantation of Pell‐1 cells in mice. (A) Photograph of an ascites‐bearing mouse injected intraperitoneally with Pell‐1 cells. (B) Cytospin preparation of the lymphoma cells in mouse ascites (May–Giemsa staining). (C) FISH analysis showing *c*‐*MYC* rearrangement (arrows) in lymphoma cells recovered from mouse ascites. (D) Photographs showing solid tumors (arrows) on multiple sites in mice. (E) Histology of the serosal membranes (hematoxylin and eosin, HE, staining). Pell‐1‐injected mouse shows a discontinuity in the mesothelial lining and increased thickness of the submesothelial region. By contrast, peritoneal tissue from a vehicle‐injected control mouse displays a uniform mesothelial cell monolayer. Tumor cells infiltrating the peritoneal membrane are shown. The tumor cells were immunostained with an anti‐CD20 antibody and counterstained with hematoxylin. (F) Histology shows tumor cells infiltrating to the serosal surface of the spleen, connective soft tissue around the kidney, and pancreatic parenchyma (HE staining). Tumor cells immunostained with the anti‐CD20 antibody are also shown

NOD/SCID mice were grouped into unirradiated and irradiated mice, and Pell‐1 cells (3 × 10^7^ per mouse) were injected intraperitoneally. The xenotransplantation experiments were performed two times independently with a total of five mice/group. These mice were sacrificed at 5 weeks postinjection. Four of five mice in the unirradiated group produced a single nodule, which was localized in the parietal peritoneum or the preperitoneal adipose tissue near the injection site. None of these mice developed visible ascites or intraperitoneal tumors. However, all irradiated mice produced profuse ascites (Figure 3A). The mean and median volumes of the ascites were 5.4 and 3.6 ml, respectively. The ascites, as well as peritoneal lavage fluids, contained numerous lymphoma cells, with mean and median total cell numbers of 3.4 × 10^8^ and 3.6 × 10^8^ cells, respectively. By contrast, the mean and median cell numbers in peritoneal lavage fluids from the unirradiated mouse group were 3.0 × 10^6^ and 0.1 × 10^6^ cells, respectively. The morphology of the lymphoma cells closely resembled that of the cultured Pell‐1 cells (Figure 3B). They had an immunophenotype identical to that of Pell‐1 cells *in vitro* and retained the same *c‐MYC* rearrangement (Figure 3C). The irradiated mice also gave rise to multiple tumors in the peritoneum, mesentery, and retroperitoneal tissues (Figure 3D). Histopathology revealed that, in contrast to a uniform monolayer of mesothelial cells in vehicle‐injected control mice, Pell‐1‐injected mice showed a discontinuity in the mesothelial lining and thickening of the submesothelial region (Figure 3E). Infiltration of tumor cells on the peritoneal membrane was observed. In addition, tumor cells invaded the serosal surface of the spleen and the fatty tissue around the kidneys, but no tumor infiltration into their parenchyma was observed (Figure 3F). All mice displayed severe pancreatic infiltration where the organ was almost entirely replaced by tumor cells. No lymph node enlargement was found in any of the mice. Also, we did not observe tumor cells invading tissues or organs in the thoracic cavity including the diaphragm or lungs. The xenotransplantation experiments were also performed using the “double‐hit” high‐grade B‐cell lymphoma cell line DH‐My6.^22^ DH‐My6 cells (3 × 10^7^ per mouse) also induced massive multiple solid tumors in all irradiated mice (*n* = 3) 5 weeks after injection, but they spread outside the abdominal cavity (data not shown). Nevertheless, ascites formation was limited (mean volume, 1.0 ml; mean cell number, 3.2 × 10^7^ cells). These findings indicate that Pell‐1 cells could grow in the peritoneal cavity and form marked effusions in this xenograft model.

**FIGURE 4:**
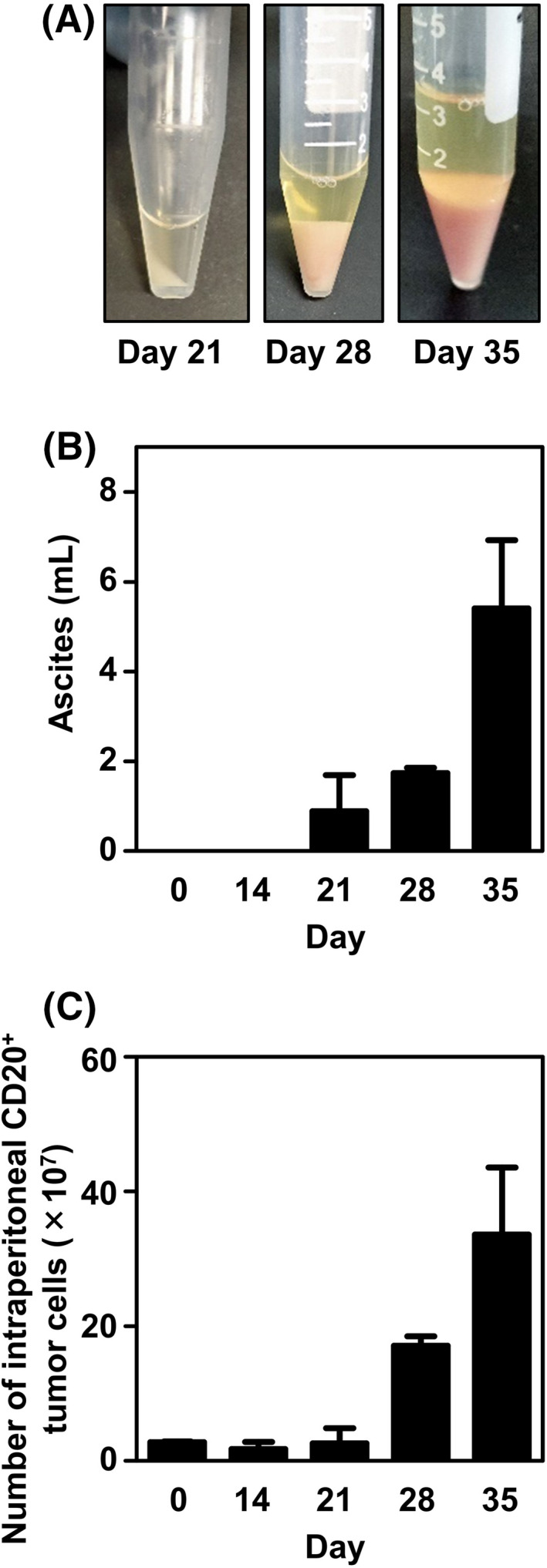
Sequential progression of lymphomatous ascites in the xenograft mouse model. Mice injected intraperitoneally with Pell‐1 cells (3 × 10^7^ per mouse) were euthanized on days 14 (*n* = 3), 21 (*n* = 3), 28 (*n* = 3), or 35 (*n* = 5) postinjection for the evaluation of ascites development. (A) Representative photographs of ascites with time. (B) The graph shows the ascites volumes measured at each time point. (C) The graph shows the numbers of lymphoma cells recovered from the ascites and peritoneal washings. The cells were stained with an anti‐CD20 antibody and counted using flow cytometry. The graphs show the mean ± standard deviation (SD; bars)

To evaluate the sequential growth of Pell‐1 cells in our xenograft mice, cells (3 × 10^7^ per mouse) were injected into 14 irradiated mice and evaluated for the occurrence of ascites and tumors on days 14, 21, 28, and 35 postgrafting (Figure 4). On day 14, no visible ascites and tumors were observed, but viable lymphoma cells could be recovered by peritoneal lavage from all mice evaluated. On day 21, notable lymphomatous ascites developed in all mice, but tumor formation was localized to the mesentery and pancreas. Thereafter, both ascites volumes and tumor cell numbers increased rapidly. By day 28, the tumors had spread to multiple sites in the peritoneal cavity and retroperitoneum. Thus, our xenograft model showed a sequential tumor progression: namely serosal spreading of lymphoma cells at the early stage and subsequent development of lymphomatous effusion, followed by solid tumor formation in the abdominal cavity at the late stage.

### 3.4 Antiproliferative activity of BET inhibitors *in vitro*

**FIGURE 5:**
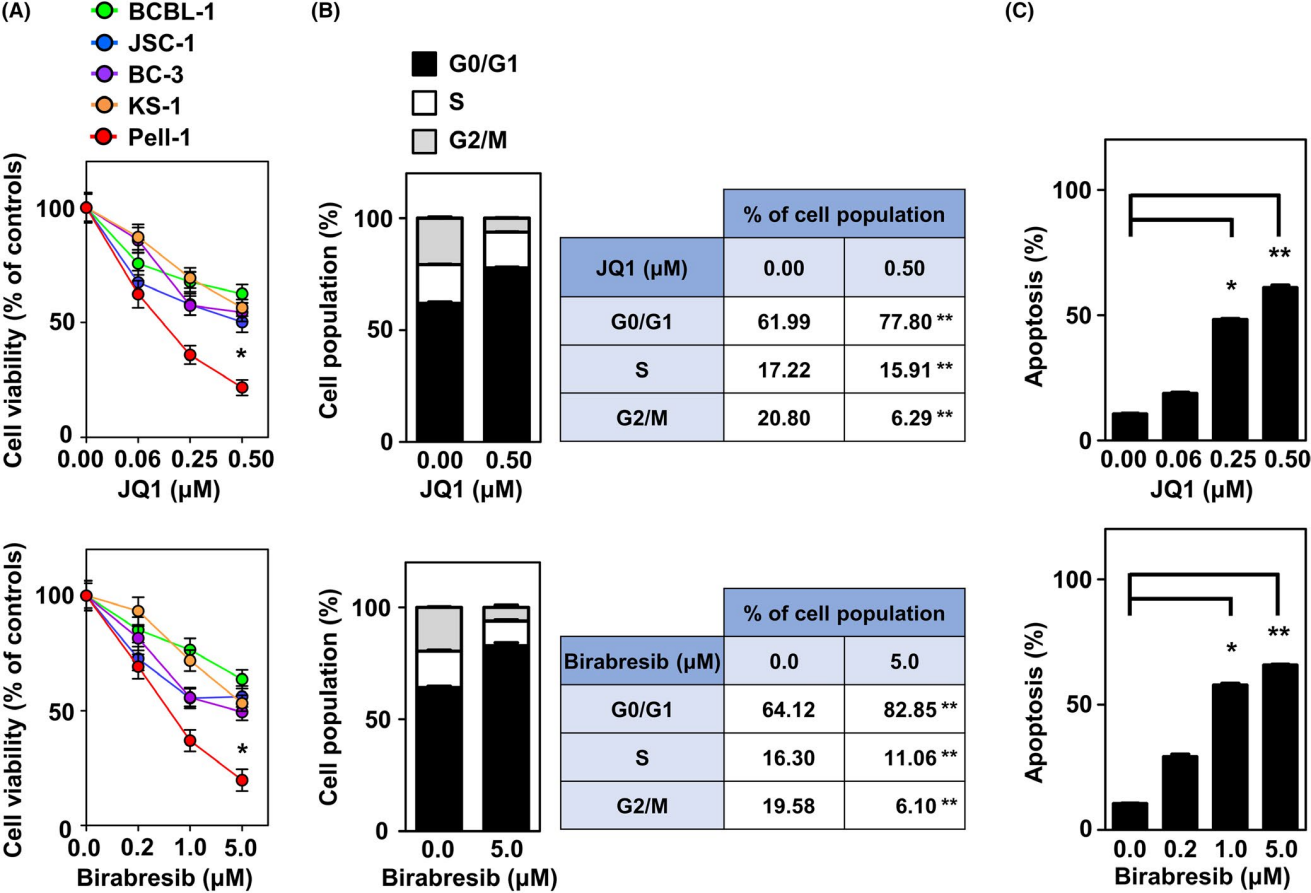
BET inhibitors suppress cell growth and induce cell cycle arrest and apoptosis *in vitro*. (A) Cell growth assays of Pell‐1 and HHV8‐positive PEL cell lines (BCBL‐1, JSC‐1, BC‐3, and KS‐1) were conducted 48 h after treatment with the indicated doses of JQ1 or birabresib. The numbers of viable cells are normalized to a percentage of the viable cell numbers of vehicle (DMSO)‐treated controls. Pell‐1 cells were significantly more sensitive to JQ1 and birabresib than all PEL cell lines tested at concentrations of 0.5 μM and 5 μM, respectively. (B) Cell cycle analysis was conducted 24 h after treatment with JQ1 (0.5 μM) or birabresib (5 μM). Percentages of the cell population in each stage of the cell cycle are presented outside the graph. (C) Apoptosis assay was conducted 48 h after treatment with the indicated doses of JQ1 or birabresib. The graphs show the percentages of apoptotic cells in the total cell population. All experiments were performed three times, and data are expressed as the mean ± SEM. Significant differences are shown as *p < 0.05, **p < 0.01

Because Pell‐1 cells had *c‐MYC* rearrangement and expressed the c‐MYC protein, we evaluated the antiproliferative effect of BET inhibitors, which suppress oncogenic transcription factors mainly through inhibiting the function of c‐MYC.^26^, ^27^ Pell‐1 cells were treated with JQ1 or birabresib at various concentrations in a medium supplemented with 20% FCS (Figure 5A). Both compounds significantly attenuated Pell‐1 cell growth in a dose‐responsive manner at 48 h, while the antiproliferative effects were significantly less for all HHV8‐positive PEL cell lines tested. The estimated half‐maximal inhibitory concentration (IC_50_) values of JQ1 and birabresib after 72 h of drug exposure were 62 nM and 114 nM, respectively, indicating high sensitivity to BET inhibition.^28^, ^29^, ^30^, ^31^ JQ1 and birabresib treatments caused cell cycle arrest at the G0/G1 phase and induced a marked increase in apoptosis (Figure 5B,C).

### 3.5 Antiproliferative activity of birabresib *in vivo*

**FIGURE 6:**
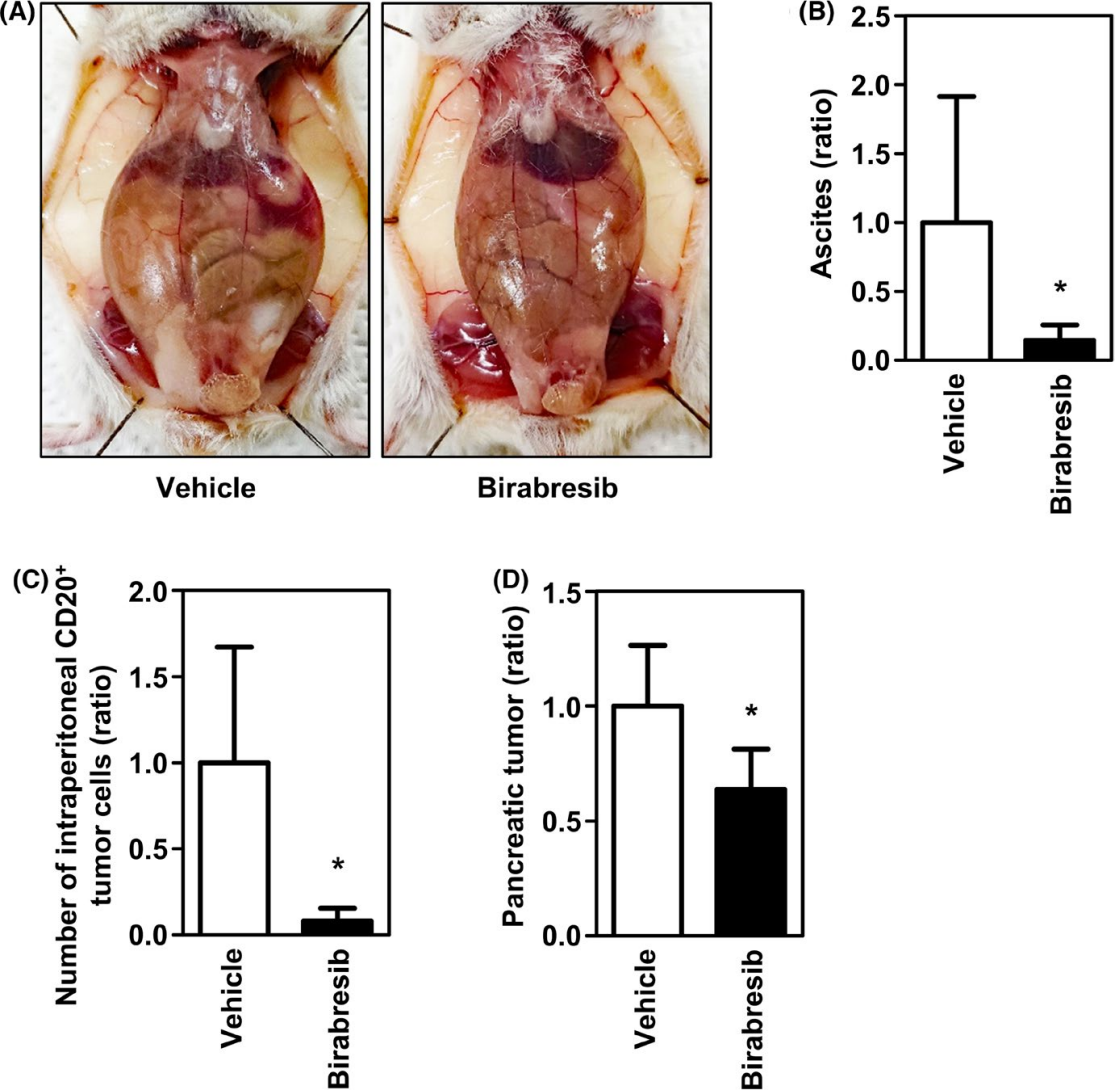
Birabresib suppresses Pell‐1 growth *in vivo*. 2 × 10^7^ Pell‐1 cells were injected into the peritoneal cavity of mice. Three days later, mice were treated with birabresib (50 mg/kg) or vehicle five days per week. Mice were euthanized on day 28. (A) Representative photographs of the mice treated with birabresib or vehicle alone. (B) The graph shows the relative ascites volumes in mice treated with birabresib versus those treated with vehicle alone. (C) The graph shows relative cell numbers of lymphoma cells between the two groups. Cells recovered from ascites and peritoneal lavage fluids were stained with an anti‐CD20 antibody. The CD20‐positive cells were counted using flow cytometry. (D) The graph shows the relative weights of tumors developing in the pancreatic region between the two groups. The data are expressed as the mean ± SD of the results obtained from six mice per group; *p < 0.05

We next evaluated the effect of BET suppression on Pell‐1 cell growth in our xenograft model (Figure 6). Irradiated mice were injected with Pell‐1 cells (2 × 10^7^ per mouse). On day 4 postinjection, mice were randomly divided into two groups and treated intraperitoneally with birabresib (50 mg/kg) or vehicle (DMSO) once daily, 5 days a week for 4 weeks. The experiments were performed twice independently with a total of six mice/group. The birabresib‐treated mice had significantly lower volumes of ascites than those of the vehicle‐treated mice (*p* = 0.002). In addition, the numbers of lymphoma cells in ascites and peritoneal lavage fluids were significantly reduced in the birabresib‐treated mouse group (*p* = 0.002). Moreover, birabresib treatment significantly suppressed the growth of the solid tumors compared with vehicle treatment (*p* = 0.026), as assessed by the weight of tumors that developed in the pancreatic region. The birabresib‐treated mice showed no signs or symptoms of toxicity such as progressive weight loss or behavioral changes indicating stress and pain. These results indicate that birabresib has antitumor activity against HHV8‐unrelated effusion large B‐cell lymphoma *in vivo*.

## 4 DISCUSSION

Here, we established a novel cell line, Pell‐1, derived from a Japanese patient with HHV8‐unrelated effusion large B‐cell lymphoma related to liver cirrhosis. Because cell line authentication has been required using an accepted consensus method, such as STR DNA profiling,^32^, ^33^, ^34^ we have provided data confirming the authenticity of Pell‐1. In addition to karyotype identity, Pell‐1 cells and the patient's primary lymphoma cells had identical DNA fingerprints in all loci employed. In this context, Pell‐1 is the first HHV8‐unrelated effusion large B‐cell lymphoma cell line whose derivation was authenticated using STR profiling. Pell‐1 cells also retained the *c‐MYC* rearrangement and immunophenotypic features of primary lymphoma cells.

While many CDX models of HHV8‐positive PEL have been generated and have contributed to the investigation of the pathogenetic role of HHV8,^35^, ^36^, ^37^, ^38^ to our knowledge, animal models of HHV8‐unrelated effusion large B‐cell lymphoma have not been established. A major strength in this study is that we established for the first time an HHV8‐unrelated effusion large B‐cell lymphoma xenograft model by injecting Pell‐1 cells into the peritoneal cavity of NOD/SCID mice, which were chosen because these immunodeficient mice have been used successfully to create CDX models of HHV8‐positive PEL.^35^, ^36^, ^37^, ^38^ Unirradiated mice injected intraperitoneally with Pell‐1 cells did not develop noticeable ascites, but viable lymphoma cells could be recovered by peritoneal lavage up to 5 weeks after injection, although the cell numbers were limited. Whole‐body irradiation has been used to promote lymphoid cell engraftment in NOD/SCID mice.^39^, ^40^ Here, we showed that irradiation of the mice led to a greater efficiency of engraftment and resulted in the production of a large volume of lymphomatous ascites and tumor masses in all xenotransplanted mice tested. These findings suggest that greater levels of immunodeficiency are required to promote the establishment of a CDX mouse model of HHV8‐unrelated effusion large B‐cell lymphoma. In keeping with this *in vivo* observation, our *in vitro* cell growth assays showed that Pell‐1 cells grew poorly at low concentrations of FCS compared with HHV8‐positive PEL cell lines. This might have accounted for the delayed growth of Pell‐1 cells in unirradiated mice, leading to early elimination of the cells.

Another strength in this study is that we demonstrated a sequential process of tumor progression in mice, which mimicked the clinical course of peritoneal HHV8‐unrelated effusion large B‐cell lymphoma. In the early stage, irradiated mice injected with Pell‐1 cells did not develop visible ascites, but peritoneal washings contained numerous lymphoma cells, indicating the proliferative capability of Pell‐1 cells in the peritoneal cavity of immunodeficient mice. It has been suggested that mesothelial cells might support HHV8‐positive PEL cell growth and survival because the proliferation of the cells was significantly increased in coculture with mesothelial cells.^41^ Our data suggest that effusion‐based lymphoma cells, irrespective of any association with HHV8 infection, can grow within a serous cavity lined with mesothelial cells in immunodeficient mice. Subsequently, the mice developed profuse ascites. Lignitto et al.^41^ observed peritoneal thickening during ascites progression in mice inoculated with the HHV8‐positive PEL cell line CRO‐AP/3. We also found thickening of the serosal membranes accompanied by lymphomatous infiltrations. These findings are consistent with radiologic studies demonstrating serosal thickening in patients with peritoneal lymphomatosis, based on computed tomography findings.^42^, ^43^ In the late stage, our experimental mice formed intraperitoneal and retroperitoneal solid tumors. We found that mesenteries and the pancreas were the most severely affected. Because the tumor cells were injected intraperitoneally, it is understandable that the mesentery was often affected. However, whether peritoneal HHV8‐unrelated effusion large B‐cell lymphoma preferentially disseminates to specific organs such as the pancreas remains unclear. Tumor mass formation likely represents the last stage of HHV8‐unrelated effusion large B‐cell lymphoma transformation. Indeed, mass formation during the clinical course of the disease is observed often in the advanced stage.^13^ Thus, our CDX model recapitulated the successive stages of HHV8‐unrelated effusion large B‐cell lymphoma progression, although a limitation was that the precise growth properties of the injected Pell‐1 cells—such as doubling time—is difficult to assay by noninvasive methods.

In contrast with HHV8‐associated PEL, *c‐MYC* or 8q24 abnormalities were found in a significant fraction of HHV8‐unrelated effusion large B‐cell lymphoma. Wu et al.^6^ reported that abnormalities of *c‐MYC* were found in 12 (48%) of 25 patients. Kaji et al.^13^ also detected *MYC* translocation in 7 (19%) of 36 cases. Thus, studies have proposed that testing for *c‐MYC*/8q24 abnormalities should be included for a differential diagnosis.^6^, ^12^, ^15^ It has also been postulated that c‐MYC expression is involved in the development of a certain proportion of HHV8‐unrelated effusion large B‐cell lymphoma cases.^6^, ^12^, ^15^ Therefore, c‐MYC could be an ideal therapeutic target for HHV8‐unrelated effusion large B‐cell lymphoma. However, largely because of the rarity of the disease, optimization of the therapeutic regimen remains elusive. In this study, we evaluated the efficacy of c‐MYC‐targeted therapy against HHV8‐unrelated effusion large B‐cell lymphoma using a Pell‐1 cell line model. Because direct anti‐c‐MYC agents are not available currently, BET inhibitors have been used as alternatives. These drugs downregulate the transcription of genes regulated by superenhancers, such as *c‐MYC*.^26^, ^27^ We showed that both JQ1 and birabresib treatments resulted in a significant attenuation of Pell‐1 cell growth *in vitro* through the induction of G0/G1 cell cycle arrest and apoptosis. Pell‐1 cells displayed significantly greater sensitivity to BET inhibitors than among all the HHV8‐positive PEL cell lines evaluated. The IC_50_ of both JQ1 and birabresib, estimated after 72 h of drug exposure, is generally lower than the IC_50_ for a panel of lymphoid cell lines, including cell lines derived from c‐MYC‐driven lymphomas such as “double‐hit” and Burkitt's lymphomas,^28^, ^29^, ^30^, ^31^ suggesting a potent antiproliferative activity of JQ1 and birabresib alone in cases of HHV8‐unrelated effusion large B‐cell lymphoma. We also validated the antitumor effect of BET inhibition *in vivo*. We chose birabresib for this study because this is the first BET inhibitor to undergo early clinical trials for hematologic tumors and solid tumors successfully in the absence of major toxicities.^44^, ^45^, ^46^ Birabresib significantly reduced ascites and inhibited lymphoma cell proliferation in the immunodeficient mouse peritoneal cavity without apparent adverse effects. In addition, this agent suppressed solid tumor progression *in vivo*. Thus, our preclinical results indicate the therapeutic potential of birabresib in cases of HHV8‐unrelated effusion large B‐cell lymphoma, although further studies are needed to evaluate whether this compound can also induce tumor regression *in vivo* as a single agent or in combination with cytotoxic agents.

In summary, we present a novel HHV8‐unrelated effusion large B‐cell lymphoma cell line, Pell‐1, with a *c‐MYC* rearrangement. The first CDX model captured the clinical phenomena observed in patients and recapitulated the successive stages of aggressive HHV8‐unrelated effusion large B‐cell lymphoma, so it provides a useful tool for the preclinical testing of new antitumor agents. Using this well‐characterized Pell‐1 model, we demonstrated that birabresib exerted potent antitumor activity both *in vitro* and *in vivo*, suggesting that such BET inhibitors are promising candidates for therapies against cases of HHV8‐unrelated effusion large B‐cell lymphoma carrying *c‐MYC* alterations. Moreover, our Pell‐1 model will allow more in‐depth studies on the pathogenesis of this unique form of lymphoma with a predilection for the East Asian population.

## CONFLICT OF INTEREST

The authors declared no potential conflicts of interest.

## Supporting information

Fig S1

Fig S2

Fig S3

## Data Availability

The data that support the findings of this study are available from the corresponding author upon reasonable request.
