# Supplementary figures and images for: Development of a novel cell line‐derived xenograft model of primary herpesvirus 8‐unrelated effusion large B‐cell lymphoma and antitumor activity of birabresib in vitro and in vivo
Source: Cancer Med. 2021 Nov 24;10(24):8976–87. doi: 10.1002/cam4.4394 (PMC8683535; doi:10.1002/cam4.4394)

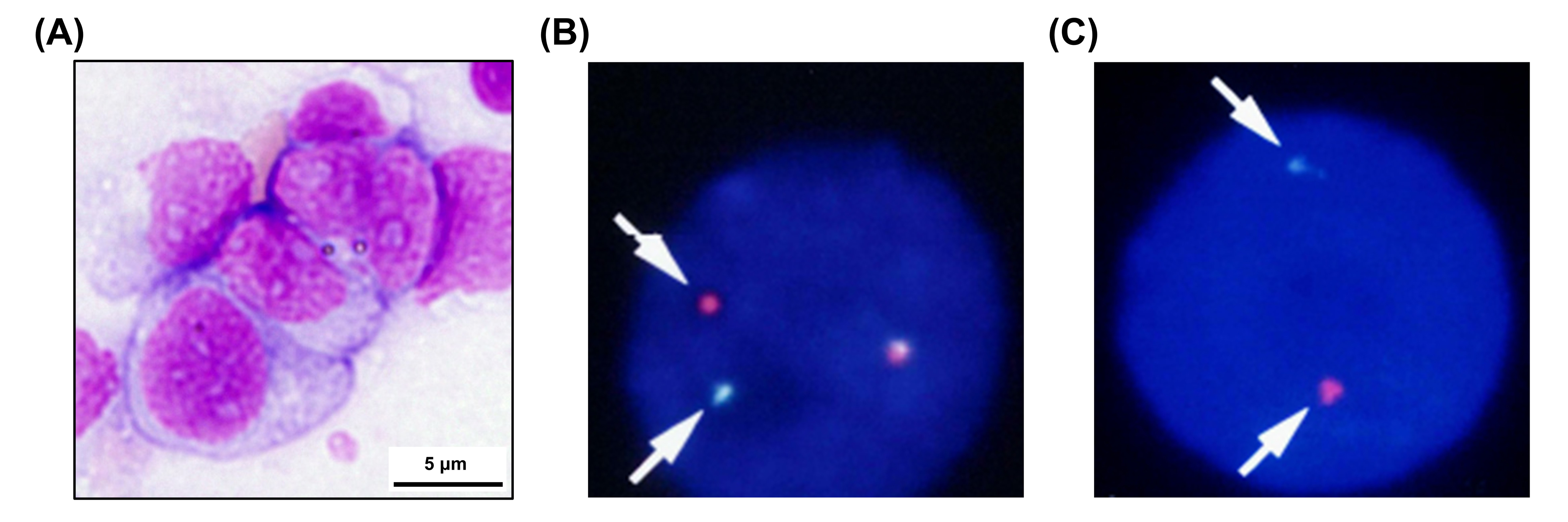

Supplement: Supplementary file 1 — Fig S1 [file CAM4-10-8976-s001.jpg]

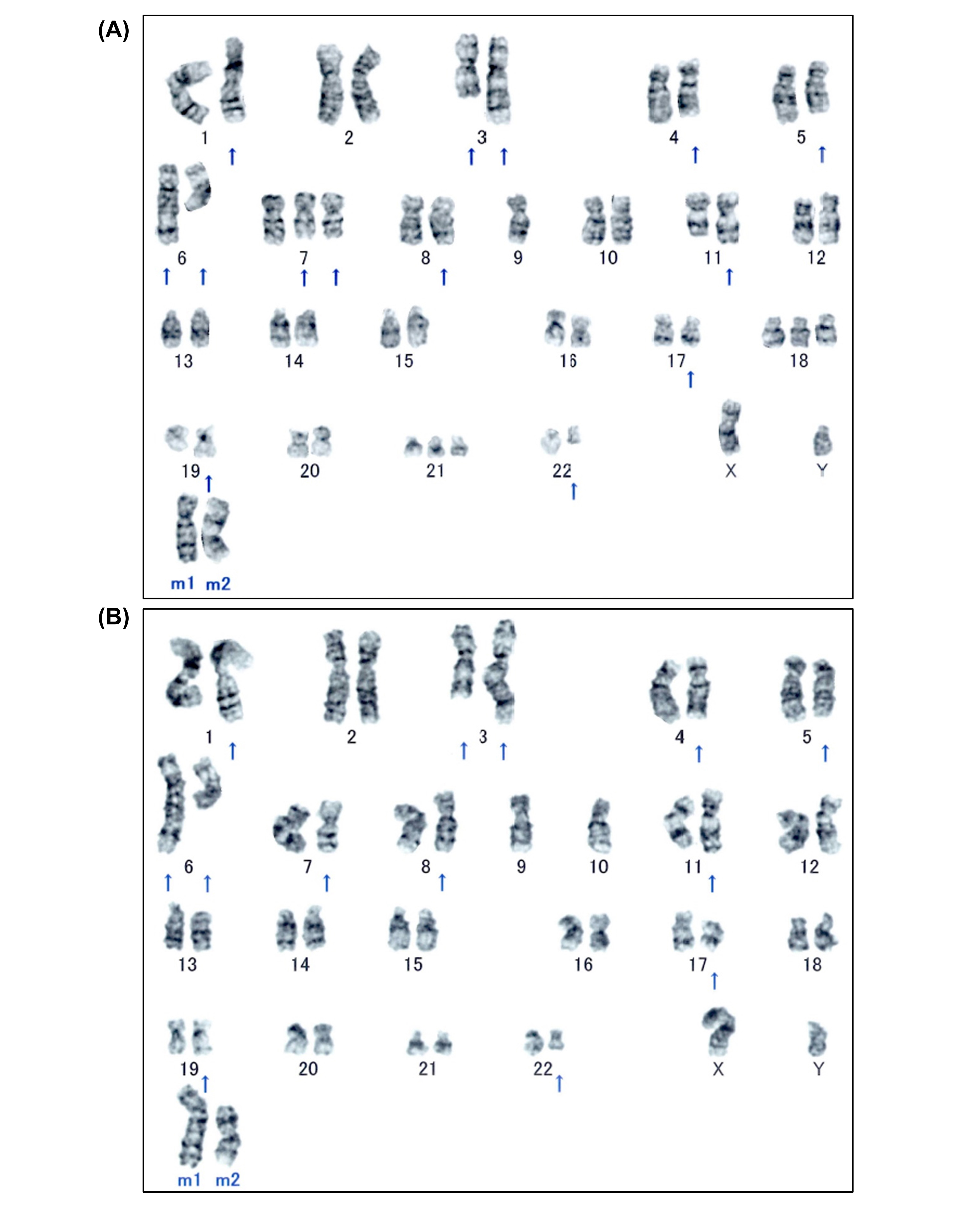

Supplement: Supplementary file 2 — Fig S2 [file CAM4-10-8976-s003.jpg]

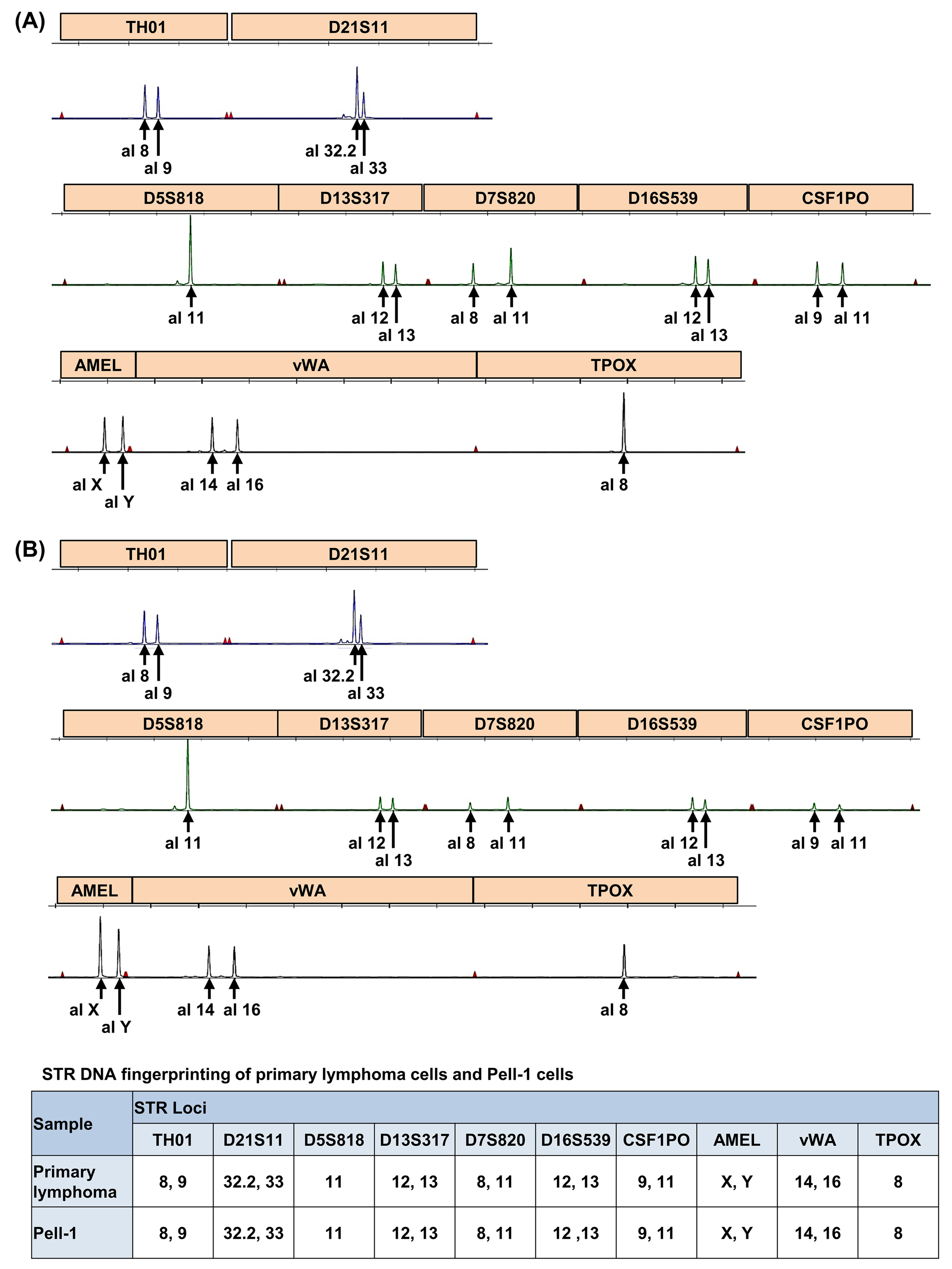

Supplement: Supplementary file 3 — Fig S3 [file CAM4-10-8976-s002.jpg]
